# The effect of previous treatment with bisphosphonate and renal impairment on the response to denosumab in osteoporosis: a ‘real-life’ study

**DOI:** 10.1007/s40618-019-01131-5

**Published:** 2019-10-29

**Authors:** T. R. Fraser, I. Flogaitis, A. E. Moore, G. Hampson

**Affiliations:** 1 Department of Chemical Pathology and Metabolic Medicine, St Thomas’ Hospital, Lambeth Palace Road, London, SE1 7EH UK; 2 Metabolic Bone Clinic, Department of Rheumatology, Guy’s Hospital, London, UK; 3 Osteoporosis Unit, Guy’s Hospital, London, SE1 9RT UK

**Keywords:** Denosumab, Bisphosphonate, Chronic kidney disease

## Abstract

**Purpose:**

To investigate changes in bone mineral density (BMD) following denosumab after previous bisphosphonate therapy and the impact of chronic kidney disease (CKD) on response.

**Methods:**

A retrospective study of 134 patients (11 M, 123 F) aged [mean (SD)] 72 [11] years on denosumab was undertaken. Ninety-five patients had previously been on oral and 28 on iv bisphosphonate. Lumbar spine (LS), total hip (TH) and femoral neck (FN) BMD were measured before treatment and at 2.7 [1.2] years. GFR was < 35 ml/min in 24 patients (18%). Ninety-four (18 M, 76 F) patients aged 71 [11] years transitioning to zoledronate were also studied.

**Results:**

BMD improved following denosumab [mean (SEM) % change LS: 6.0 (0.62) *p* < 0.001, TH: 2.28 (0.64) *p* < 0.001, FN: 1.9 (0.77) *p* = 0.045]. Changes at the TH and FN were lower in patients with GFR < 35 ml/min (Group B) compared to those with GFR > 35 ml/min (Group A) [% change TH; Group A: 2.9 (0.72), Group B: − 0.84 (1.28), *p* = 0.015, FN; Group A: 2.76 (0.86), Group B: − 1.47 (1.53), *p* = 0.025]. % change in BMD at the FN and PTH were negatively associated (*r* = − 0.25, *p* = 0.013). BMD changes were not different at 12–18 months between patients on denosumab compared to zoledronate [% change at LS: denosumab: 3.97% (0.85), zoledronate: 2.6% (0.5), *p* = 0.19 TH: denosumab: 0.97% (0.58), zoledronate: 0.92% (0.6), *p* = 0.95).

**Conclusion:**

Denosumab increases BMD following previous bisphosphonate treatment and is comparable to zoledronate. Lower response seen at the hip in CKD is related to PTH concentrations.

## Introduction

Denosumab is a monoclonal antibody that binds and inhibits RANKL, resulting in suppression of bone resorption. It is a potent anti-resorptive agent used in the treatment of osteoporosis. The phase 3 pivotal FREEDOM trial (Fracture REduction Evaluation of Denosumab in Osteoporosis Every 6 Months) showed its anti-fracture efficacy at all skeletal sites [1]. A 68% reduction for vertebral fractures (VFs), 20% for non-VFs and 40% for hip fractures compared with placebo were observed after 36-month treatment. Bone mineral density increased progressively, reaching an overall increase of 6% and 9.2% at the LS and 4% and 6.0% at the TH compared to placebo at 24 and 36 months, respectively.

However, patients participating in the FREEDOM trial were treatment naïve or had been off treatment with bisphosphonates (BPs) for at least 12 months. A few short-term randomised clinical trials evaluating the effects of previous treatment with BPs, which have long skeletal retention time, on the response to denosumab have been carried out. These studies have looked and compared surrogate endpoints such as changes in BMD or bone turnover markers. Greater increases in BMD and reduction in bone turnover were seen in patients transitioning to denosumab compared to those who continued oral BPs (alendronate or risedronate), although the increases in BMD were smaller than those seen in treatment-naïve patients [2–5]. Few studies have been done in ‘real-life setting’ where most patients transitioning to denosumab will have had previous exposure to BPs. In the UK, in particular, the National Institute for Health Care Excellence (NICE) recommends denosumab as a treatment option for the prevention of osteoporotic fragility fractures in postmenopausal women at increased risk of fractures who are unable to comply with specific dosing protocol for oral bisphosphonate or are intolerant or have a contraindication to those treatments (TA204, https://bnf.nice.org.uk/drug/denosumab.html).

BPs are indeed contra-indicated in patients with chronic kidney disease (CKD), particularly those with GFR < 35 ml/min (in the case of alendronate) or < 30 ml/min (in the case of risedronate). Denosumab, however, can be used in patients who have osteoporosis and CKD, although there is an increased risk of hypocalcaemia in patients with severe renal impairment or end-stage renal disease [5–7]. Post hoc analysis of data from the FREEDOM trial showed that denosumab was still effective in terms of fracture reduction and improvement in BMD in patients with CKD stage 3 and 4 [8]. However, none of the patients in the trial had secondary hyperparathyroidism. It has been previously shown that hyperparathyroidism at baseline can impair the response to alendronate, an oral bisphosphonate [9]. However, it is unclear whether hyperparathyroidism would have an impact on the response to denosumab in ‘real-life’.

There is thus a paucity of information as to the efficacy of denosumab in the context of routine clinical practice in addressing the issues above which are namely, previous exposure to BPs and patients with osteoporosis and renal impairment. The aim of the current study was to investigate changes in BMD following denosumab in patients who have been on previous osteoporosis drugs (mainly BPs) to determine whether this has an impact on the response to denosumab and to compare changes in BMD in patients transitioning to denosumab or zoledronate to determine whether denosumab therapy increases bone mineral density (BMD) more so than zoledronate. We also reviewed whether the response to denosumab is affected by renal impairment (GFR < 35 ml/min) in the real-life setting of a metabolic bone clinic.

## Materials and methods

### Subjects

We carried out a retrospective study of 134 patients (11M, 123F) aged [mean (SD)] 72 [11] years attending the metabolic bone clinic who had started treatment with denosumab between April 2013 and February 2017 for osteoporosis following treatment with bisphosphonates. They were included in the study as they had received denosumab for at least 12 months. Clinical information, BMD and biochemical measurements were obtained during their follow-up appointments. We also analysed data from 94 patients (76F and 18M) with a mean age of 71 [11] years with similar clinical characteristics to the denosumab cohort in terms of fracture risk who had transitioned to iv zoledronate from oral bisphosphonate to compare the efficacy of the 2 parenteral osteoporosis drugs.

All patients were advised to continue vitamin D (800 IU daily) and calcium supplements (if their dietary calcium was < 700 mg/daily). Patients with 25(OH)vitamin D < 35 nmol/L were given additional cholecalciferol to increase 25(OH)vitamin D concentrations above 50 nmol/L prior to treatment. They were also advised to have their serum calcium measured within 2 weeks following the denosumab injection.

Data collection was done using the hospital’s electronic patient record system and medical records. This was closely supervised by the senior clinical team as part of service review and complied with the UK data protection act in accordance with the institution research and development committee. All data were anonymised. BMD data at the lumbar spine (LS), femoral neck (FN) and total hip (TH) were obtained prior to starting treatment with denosumab (baseline values) and compared with values at a mean [SD] of 2.7 [1.2] years following treatment. Baseline and follow-up BMD were measured on the same bone densitometry scanner. Percentage changes in BMD at the LS, FN, and TH were derived following treatment with a mean [SD] duration of 2.7 [1.2] years and compared to pre-treatment values in 134, 110 and 123 patients, respectively. Changes in BMD were derived after 12 months in the group on zoledronate and compared with a sub-group of patients on denosumab (*n* = 27) who had a BMD measurement at 12–18 months after treatment. All scans were performed by certified technologists who are trained in the measurement and interpretation of BMD. Routine laboratory measurements included estimated glomerular filtration rate (eGFR), albumin-corrected calcium (ACC), PTH concentration, and 25(OH)vitamin D that were done prior to the treatment. Patients demographics, BMD and biochemical data are summarised in Table 1.

**Table 1:** Summary of the demographics, BMD and biochemical parameters of the study population

Parameter; mean (SD)	Denosumab	Zoledronate
No. of participants [M/F]	134 [11 M/123 F]	94 [18 M/76 F]
Age (years)	72 (11)	71 (11)
BMD lumbar spine (g/cm^2^)	0.790 (.135)	0.798 (0.13)
‘T’ score lumbar spine	− 2.4 (1.2)	− 2.3 (1.2)
BMD total hip (g/cm^2^)	0.687 (0.107)	0.68 (0.12)
‘T’ score total hip	− 2.1 (0.8)	− 2.2 (0.9)
BMD femoral neck (g/cm^2^)	0.584 (0.1)	0.577(0.1)
‘T’ score femoral neck	− 2.2 (0.9)	− 2.5 (0.9)
Previous fractures (%)	113 (84%)	59 (63%)
eGFR (ml/min)	64 (27)	80 (19)
PTH (ng/l)	55 (37)	41 (17)
Corrected calcium (mmol/l)	2.4 (0.1)	2.3 (0.1)
25 (OH)Vitamin D (nmol/l)	72 (28)	63 (29)

### Measurement of bone mineral density (BMD)

BMD at the LS, FN and TH was measured by dual-energy X-ray absorptiometry (DXA) (Hologic QDR 4500A; Hologic, Inc., Bedford, MA, USA). The precision error for measurement of BMD at the spine and total hip was 1.0% and 1.2%, respectively.

### Laboratory measurements

Routine laboratory tests were determined using standard laboratory methods on the Roche analysers (Roche diagnostics Limited, West Sussex, UK). eGFR was calculated using the Modification of Diet in Renal Disease formula [10]. Assay CVs for serum intact PTH were < 5% at PTH concentrations of 41 and 105 ng/L. Serum 25-hydroxyvitamin D (25(OH)vitamin D) assay was performed using an automated immunoassay on the Abbott Architect (Abbott Laboratories, Abbott Park, Illinois, USA). Assay CVs ranged between 5.0 and 10.7% at serum concentrations between 25 and 85 nmol/L.

### Statistical analysis

Statistical analysis was performed using IBM SPSS Statistics 24 for Windows (LEADTOOLS©, LEAD Technologies, Inc., USA). Mean and SD were derived for all continuous variables. The paired student *t* test was performed to compare baseline and follow-up values. Unpaired *t* test was used to compare means between two groups. Pearson correlation was used to explore the relationship between continuous variables. Multi-linear regression analyses were carried out to determine the association between % change in BMD with PTH concentrations following adjustment for possible confounders including the presence of secondary risk factors. A “*p*” value < 0.05 was considered statistically significant.

## Results

### Clinical characteristics of study subjects

Of the 134 patients receiving denosumab, 127 (95%) had sustained one or more fragility fractures. Fifty patients had a secondary risk factor for osteoporosis: 34 patients were either on or had previous exposure to glucocorticoids, rheumatoid arthritis (*n* = 8), systemic lupus erythematosus (SLE) (*n* = 3), endocrine disorders (primary hyperparathyroidism/diabetes mellitus) *n* = 2 and treatment with aromatase inhibitors (*n* = 7). One hundred and twenty-three (92%) patients had previous treatment with bisphosphonates for 6.4 (4.5) years including 28 on iv zoledronate and 95 on oral bisphosphonates. Patients were transitioned to denosumab, at least 12 months following their last dose of zoledronate. Eleven patients were not on bisphosphonate because of renal impairment. Reasons for changing to denosumab included lack of efficacy in those with GFR > 35 ml/min (*n* = 50), intolerance and/or contra-indications (*n* = 84) which included 24 patients with GFR < 35 ml/min. None of the patients sustained any new fractures whilst taking denosumab. Only one patient stopped treatment within 12 months (after the first dose) because of skin rash.

Patients on denosumab were divided into 2 groups based on their eGFR; Group A (*n* = 105) with eGFR > 35 ml/min and Group B (*n* = 24) with eGFR < 35 ml/min when bisphosphonates are contra-indicated. There were no differences in age and pre-treatment BMD between the two groups. However, there were statistically significant differences in biochemical parameters; (Group A; mean [SD] PTH: 46 [20] Group B; 99 [62] ng/L, *p* < 0.001) and 25 (OH)vitamin D (Group A; 75 [28] Group B; 56 [29] nmol/L *p* = 0.017). The data are summarised in Table 2.

**Table 2:** Summary of demographics, BMD and biochemical parameters in patients with GFR < 35 ml/min and GFR > 35 ml/min

Parameter (mean [SD])	Group A (eGFR > 35)	Group B (eGFR < 35)
No. M/F	4 M/105 F	7 M/17 F
Age (years)	72 (12)	73 (10)
BMD lumbar spine (g/cm^2^)	0.78 (0.12)	0.84 (0.17)
T-score lumbar spine	− 2.4 (1.2)	− 2.00 (1.5)
BMD total hip (g/cm^2^)	0.68 (0.11)	0.71 (0.10)
T-score total hip	− 2.1 (0.9)	− 2.00 (0.8)
BMD femoral neck	0.58 (0.10)	0.58 (0.09)
T-score FN	− 2.4 (0.9)	− 2.5 (0.8)
eGFR (ml/min)	72 (22)	26 (6)**
PTH (ng/l)	46 (20)	99 (62)**
Corrected calcium (mmol/l)	2.4 (0.1)	2.4 (0.1)
Vitamin D (nmol/l)	75 (28)	56 (29)*

**p < 0.001, *p = 0.017

All patients (*n* = 94) transitioning to zoledronate (*n* = 94) had been on previous oral bisphosphonates for 4.3 [3.3] years. Sixty-nine (74%) had sustained one or more fragility fractures. Clinical indications for transitioning were similar to the denosumab cohort and included intolerance/contra-indications due to upper gastro-intestinal side effects (*n* = 44), history of reflux disease or Barrett’s oesophagus or poor response to oral bisphosphonates (*n* = 50). Thirty-nine patients had a secondary risk factor for osteoporosis: 21 patients were either on or had previous exposure to glucocorticoids, rheumatoid arthritis (*n* = 5), polymyalgia rheumatica (*n* = 3), systemic lupus erythematosus (SLE) (*n* = 5), inflammatory bowel disease (*n* = 3), endocrine disorders (primary hyperparathyroidism/diabetes mellitus) *n* = 5, and treatment with aromatase inhibitors (*n* = 2). They were matched for age, baseline BMD and biochemical parameters. None of them had CKD stage 4 unlike the denosumab cohort.

### Changes in BMD following denosumab

**Fig. 1:**
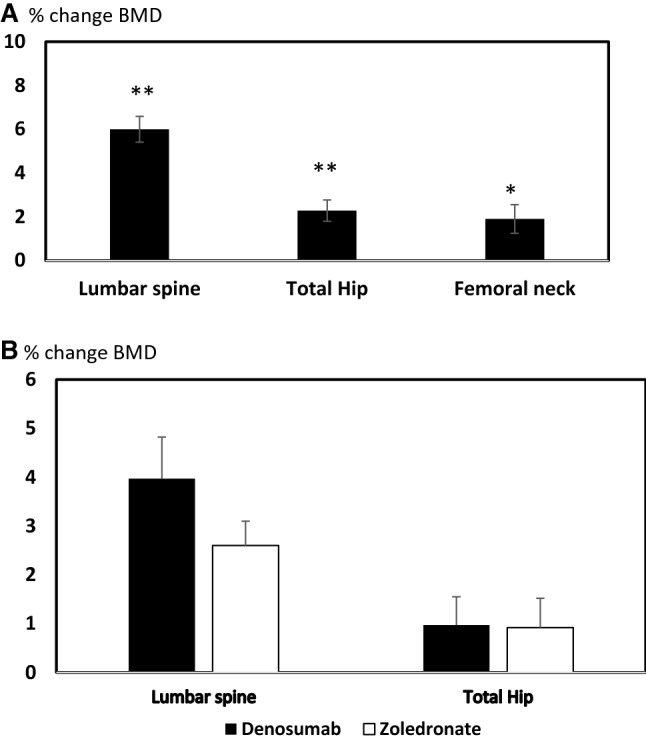
**a** Percentage change in BMD in the lumbar spine, total hip and femoral neck in the group of patients transitioning to denosumab, **b** change in BMD at the lumbar spine and total hip in patients transitioning to iv zoledronate compared to those transitioning to denosumab. There was no significant difference between the two groups. Error bars denote the standard error of the mean (SEM). *p = 0.045, **p < 0.001

A significant increase in BMD was seen at all three sites in the group receiving denosumab as illustrated in Fig. 1. The mean [SEM] % change in BMD at the LS, TH and FN were 6% [0.62], *p *< 0.001, 2.3% [0.64], *p* < 0.001 and 1.9% [0.77], *p* < 0.01, respectively. We did not observe a significant difference in % change in BMD between patients previously on iv zoledronate compared to oral bisphosphonate (mean [SEM]  % change BMD LS; zoledronate: 5.9% [1.0], oral bisphosphonate: 6.3% [0.77] *p* = 0.8, TH; zoledronate: 1.0% [0.81], oral bisphosphonate: 3.2% [0.83], *p* = 0.07, FN; zoledronate − 0.5% [1.25], oral bisphosphonate: 2.5% [0.91], *p* = 0.08), although there was a trend at the hip sites where the response to denosumab was less in patients on previous iv zoledronate transitioning to denosumab. There was no significant correlation between the length of previous exposure to bisphosphonates and % change in BMD at the LS, TH and FN following denosumab (LS: *r* = 0.02, *p* = 0.8, TH: *r* = 0.01, *p* = 0.9, FN: *r* = 0.01, *p* = 0.9).

There were no significant differences in % change in BMD at 12–18 months in the group on zoledronate compared to the group receiving denosumab (% change at LS (mean [SEM]: denosumab: 3.97% [0.85], zoledronate: 2.6% [0.5], *p *= 0.19 TH: denosumab: 0.97% [0.58], zoledronate: 0.92% [0.6] *p *= 0.95).

**Fig. 2:**
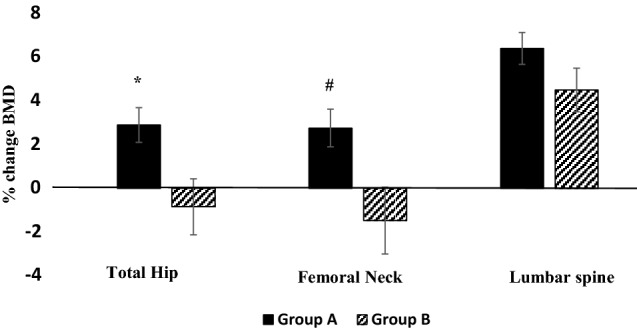
Percentage change in BMD at total hip (TH), femoral neck (FN) and lumbar spine (LS) in patients with CKD (Group B) and without CKD (Group A). Error bars denote the SEM. Changes in BMD were significantly lower at the hip sites in patients with GFR < 35 ml/min (Group B). *p = 0.015, ^#^*p* = 0.025

Changes in BMD were significantly lower at the hip sites in Group B on denosumab (patients with GFR < 35 ml/min) compared to Group A (*p* < 0.05) as shown in Fig. 2. No significant differences were observed at the lumbar spine between the two groups (mean [SEM] % change in BMD at LS; Group A: 6.4% [0.73], Group B: 4.5% [1.0], *p* = 0.15).

### Association between pre-treatment PTH concentration and % change in BMD

To further understand the differences in response to denosumab between the two groups, we examined the association between the biochemical parameters and % change in BMD. PTH concentration was available in 123 patients before treatment with denosumab was initiated and paired results were available in 94 patients (79%) following treatment for > 12 months and included 15 out of the 24 patients (63%) with GFR < 35 ml/min. A significant increase in serum PTH was observed following treatment > 12 months (mean [SD] PTH; pre-treatment: 53.6 [35] ng/L, post-treatment: 66.2 [71], *p *= 0.045). There was no difference in % change in PTH between patients with GFR < 35 ml/min and those with GFR > 35 ml/min (% change in PTH, mean [SD], Group B: 42 [84], Group A: 20[57], *p* = 0.4). We onserved a significant decrease in GFR in the whole study population over time (mean [SD] *n* = 113 baseline: 62.4 [27], post-treatment > 12 months: 58.5 [27] ml/min, *p* < 0.01). There was no reduction in albumin-corrected calcium.

There was a significant negative correlation between PTH and % change in BMD at the FN in the group who transitioned to denosumab (− 0.25, *p* = 0.013). This remained significant following correction for age (model 1), *p* = 0.014, eGFR (model 2), *p* = 0.048, presence of secondary risk factors which included current or previous treatment with glucocorticoids, inflammatory diseases (rheumatoid arthritis, systemic lupus erythematosus SLE), endocrine disorders (primary hyperparathyroidism, diabetes mellitus) and CKD (model 3), *p* = 0.018. This is shown in Table 3. There was no significant correlation with PTH and % change at LS (*r *= − 0.06, *p* = 0.53) and TH (*r *= − 0.18, *p* = 0.06). We observed a significant negative correlation between baseline PTH and % change at the TH (*r* = − 0.29, *p* = 0.008) but not at the LS (*r* = − 0.18, *p* = 0.09) in the group who transitioned to iv zoledronate.

**Table 3:** The association between BMD response to denosumab at the femoral neck (FN) and serum PTH concentrations

	Unstandardized coefficients		Standardised coefficients	*t*	Sig.
	*B*	Standard error	Beta		
Model 1					
Baseline PTH (ng/L)	**− 0.057**	**0.023**	**− 0.246**	**− 2.496**	**0.014**
Age (years)	0.055	0.074	0.074	0.746	0.458
Model 2					
Baseline PTH (ng/L)	**− 0.049**	**0.024**	**− 0.212**	**− 2.0**	**0.048**
Baseline eGFR (ml/min)	0.032	0.032	0.104	0.980	0.330
Model 3					
Baseline PTH (ng/L)	**− 0.058**	**0.024**	**− 0.252**	**− 2.409**	**0.018**
Presence of secondary risk factors	0.108	1.769	0.006	0.061	0.952

Dependent variable: % change in FN BMD following treatment with denosumab

Significant values are shown in bold

## Discussion

Our data show that treatment with denosumab in ‘real-life’ setting leads to improvement in BMD in patients previously treated with bisphosphonates and is comparable to published data in treatment-naïve patients [8]. There was no significant difference in changes in BMD at 12 months in patients who switched to denosumab compared to iv zoledronate. The skeletal response in patients with CKD stage 4 is significantly lower at the hip. A negative independent association between the response to denosumab at the FN and PTH concentrations was observed.

Smaller changes in BMD with denosumab occur following previous exposure to bisphosphonate compared with bisphosphonate treatment-naïve patients [2–4]. This may be related to reduced remodelling space which occurs during treatment with bisphosphonate. In our study population, we observed similar increases in BMD at the LS, TH and FN as previously reported in the FREEDOM trial, despite previous exposure to bisphosphonate and irrespective of duration of treatment with bisphosphonates. Indeed, several studies have shown comparable changes in BMD at the lumbar spine in treatment-naïve patients or those previously treated with iv zoledronate [11, 12]. The response to denosumab seen in our study may be, in part, related to sub-optimal adherence to oral bisphosphonate in real life or to more pronounced suppression of bone resorption by denosumab compared to oral bisphosphonate with a balance favouring modelling-based bone formation with denosumab, particularly at skeletal sites with a high content of cortical bone [13]. In contrast, the reduced response at the TH and FN which we observed in patients transitioning from iv zoledronate to denosumab can be attributed to zoledronate’s higher anti-resorptive potency, skeletal retention time and better adherence compared to oral bisphosphonate leading to a more pronounced reduction in the bone remodelling space. The changes in BMD following treatment with denosumab compared to zoledronate at 12 months were similar at the hip. Our data are in contrast to a previous RCT in post-menopausal osteoporosis where larger increases were found following denosumab at all skeletal sites after transitioning from oral bisphosphonates [4], although the discrepant findings may be explained by the smaller sample size and differences in clinical setting and population. There are no data regarding the anti-fracture efficacy of patients transitioning to denosumab from bisphosphonates. However, in the FREEDOM trial, changes in BMD have been associated with a reduced fracture incidence in post-menopausal osteoporosis [1]. We can thus extrapolate the anti-fracture efficacy across a study population with similar fracture risk.

Denosumab can be given to patients with GFR < 30 ml/min. However, as it is an anti-resorptive agent, it can further suppress bone remodelling in patients with CKD stage 4–5 who have adynamic bone disease (ABD) and can potentially be harmful. Thus, careful consideration is required before therapeutic decision making [14]. In post hoc sub-group analysis of the FREEDOM trial, significant reduction in vertebral fractures was seen in patients with CKD stage 3 but not in CKD stage 4, due to lack of statistical power [8]. However, BMD response was similar in patients with CKD stage 4 compared to CKD stage 3 at the TH and FN, although failed to reach significance at the LS. We observed a significant increase in BMD at the LS in patients with CKD 4, although % change in BMD at the TH and FN was significantly lower. One explanation for our findings is higher PTH concentrations in patients with CKD stage 4 compared to participants in the FREEDOM trial where PTH was normal.

Secondary hyperparathyroidism in CKD and end-stage renal disease is known to lead to bone loss, in particular cortical bone, and increased fracture risk [15]. Our study shows that the response to denosumab at the FN is negatively associated with serum PTH. Exposure to elevated PTH concentration, particularly if long term, may explain the reduced response to denosumab in patients with eGFR < 35 ml/min as it can lead to high bone remodelling and counteract the anti-resorptive effect of denosumab. Thus, in patients with high PTH, decreasing PTH may improve response and reduce fracture risk [16, 17]. Indeed, treatment of hyperparathyroidism with calcitriol has been shown to improve BMD response to alendronate [9]. There is, however, a lack of data on the use of denosumab in hyperparathyroidism. Reducing or reversing the biochemical abnormalities including secondary hyperparathyroidism in patients with CKD stage 4 and osteoporosis before starting specific anti-fracture agent is recommended [17]. PTH concentrations have also been shown to increase following administration of denosumab [18]. This is supported by our findings. The compensatory increase in PTH may occur to maintain calcium homeostasis as a result of denosumab-induced decrease in serum calcium due to its anti-resorptive effect.

Optimal circulating 25(OH)vitamin D level in CKD that protects against bone loss and fractures is controversial [19]. For non-dialysis CKD patients, the 2017 KDIGO guidelines recommend the same cutoff as for the general population [17]. Several studies have suggested a higher cutoff value in CKD patients might be necessary to prevent fractures and improve PTH and bone turnover [20, 21]. Increasing serum 25(OH)vitamin D levels has been shown to be associated with decreased bone turnover although it is still unclear whether correction of vitamin D status in this population decreases fracture risk [22, 23]. The lower 25(OH)vitamin D concentration at baseline in the sub-group with eGFR < 35 ml/min is unlikely to have contributed to the reduced BMD response in our patients with CKD as they were all prescribed vitamin D supplements. Our data suggest that, in addition, active vitamin D or vitamin D receptor agonist therapy may be required or adjusted in patients already receiving vitamin D analogs to control PTH concentrations as previously described [24, 25] before starting denosumab to maximise its skeletal benefits as well as preventing the early occurrence of hypocalcaemia.

Previous exposure to bisphosphonates, particularly oral bisphosphonate, and the presence of secondary clinical risk factors in a population attending a routine metabolic bone clinic does not affect the BMD response to denosumab. The effect of denosumab is lower at the hip sites in patients with CKD stage 4. This study, however, has certain limitations. First, the study is retrospective and we do not have a treatment-naïve group as control. The number of patients with CKD is small. BMD evaluation after denosumab was not done at fixed time points as would be in a randomised controlled trial design. We did not have data on routine biochemical markers of bone turnover which could have provided additional information. Irrespective of those limitations, our study provides important information about the response to denosumab in real-life setting where it is used as second-line treatment following treatment failure or contra-indications to bisphosphonate. Furthermore, our study suggests that elevated PTH concentrations may impair the response to denosumab at the hip sites in patients with CKD. Future studies are needed to investigate if maintaining PTH within the normal range in this population may improve outcome.

## Data Availability

The data that support the findings of this study are available from the corresponding author, [GH], upon reasonable request.
